# Immunological study of COVID-19 vaccine candidate based on recombinant spike trimer protein from different SARS-CoV-2 variants of concern

**DOI:** 10.3389/fimmu.2022.1020159

**Published:** 2022-09-29

**Authors:** Erika Rudi, Pablo Martin Aispuro, Eugenia Zurita, Maria M. Gonzalez Lopez Ledesma, Daniela Bottero, Juan Malito, Magali Gabrielli, Emilia Gaillard, Matthew Stuible, Yves Durocher, Andrea V. Gamarnik, Andrés Wigdorovitz, Daniela Hozbor

**Affiliations:** ^1^ Laboratorio VacSal, Instituto de Biotecnología y Biología Molecular (IBBM), Facultad de Ciencias Exactas, Universidad Nacional de La Plata, Centro Científico Tecnológico – Consejo Nacional de Investigaciones Científicas y Técnicas (CCT-CONICET), La Plata, Argentina; ^2^ Fundación Instituto Leloir-IIBBA CONICET, Buenos Aires, Argentina; ^3^ INCUINTA INTA, CONICET, HURLINGHAM, INTA Castelar, Buenos Aires, Argentina; ^4^ Human Health Therapeutics Research Center, National Research Council Canada, Montreal, QC, Canada

**Keywords:** COVID-19, SARS-CoV-2, mice immunization, spike trimeric protein, immunogenicity, vaccine candidate

## Abstract

The emergency of new SARS-CoV-2 variants that feature increased immune escape marks an urgent demand for better vaccines that will provide broader immunogenicity. Here, we evaluated the immunogenic capacity of vaccine candidates based on the recombinant trimeric spike protein (S) of different SARS-CoV-2 variants of concern (VOC), including the ancestral Wuhan, Beta and Delta viruses. In particular, we assessed formulations containing either single or combined S protein variants. Our study shows that the formulation containing the single S protein from the ancestral Wuhan virus at a concentration of 2µg (SW2-Vac 2µg) displayed in the mouse model the highest IgG antibody levels against all the three (Wuhan, Beta, and Delta) SARS-CoV-2 S protein variants tested. In addition, this formulation induced significantly higher neutralizing antibody titers against the three viral variants when compared with authorized Gam-COVID-Vac-rAd26/rAd5 (Sputnik V) or ChAdOx1 (AstraZeneca) vaccines. SW2-Vac 2µg was also able to induce IFN-gamma and IL-17, memory CD4 populations and follicular T cells. Used as a booster dose for schedules performed with different authorized vaccines, SW2-Vac 2µg vaccine candidate also induced higher levels of total IgG and IgG isotypes against S protein from different SARS-CoV-2 variants in comparison with those observed with homologous 3-dose schedule of Sputnik V or AstraZeneca. Moreover, SW2-Vac 2µg booster induced broadly strong neutralizing antibody levels against the three tested SARS-CoV-2 variants. SW2-Vac 2µg booster also induced CD4+ central memory, CD4+ effector and CD8+ populations. Overall, the results demonstrate that SW2-Vac 2 µg is a promising formulation for the development of a next generation COVID-19 vaccine.

## Introduction

On 31 December 2019, pneumonia cases of unknown aetiology with a common source of exposure at Wuhan’s South China Seafood market were reported. Later a novel coronavirus (SARS-CoV-2) was identified as the causative agent of respiratory symptoms for these cases. The outbreak rapidly evolved, affecting other countries worldwide and on 11 March 2020, the Director-General of WHO declared the COVID-19 outbreak a pandemic. Globally, as 5 August 2022 there have been 579,092,623 confirmed cases of COVID-19, including 6,407,556 deaths (https://worldhealthorg.shinyapps.io/covid/). The COVID-19 devastating impact over the past 2.5 years has resulted in a global effort to establish high level of protection by vaccination (1). Since the end of 2020, COVID-19 vaccines mainly targeting the spike protein (S), such as messenger RNA (Moderna - mRNA-1273mRNA; Pfizer-BioNTech Comirnaty - BNT162b2), protein-based (Novavax - NVX-CoV2373, Soberana 02, Abadala, Sobena Plus), viral vectored (Sputnik V - Gam-COVID-Vac-rAd26/rAd5, Oxford-AstraZeneca - AZD1222/ChAdOx1, Johnson & Johnson Janssen - Ad26.COV2.S, CANSINO- Ad5.COV2) or inactivated (Sinopharm - BBIBP-CorV, Sinovac - CoronaVac, Covaxin - BBV152) that target the entire virus, began to be authorized for use in the health emergency (2). Active immunization with these COVID-19 vaccines has significantly reduced the most negative effects of this disease, that is, hospitalizations, deaths, and even the prevalence of sequelae (3–5). As of 5 August 2022, 67.2% of the world population has received at least one dose of a COVID-19 vaccine and 12.39 billion doses have been administered globally. Though these numbers are positively impressive, a worrying fact is the inequity in the distribution of vaccines, only 20.2% of people in low-income countries have received at least one dose (Data OWi. Coronavirus (COVID-19) Vaccinations. Oxford, United Kingdom: University of Oxford; (2022). Available at: https://ourworldindata.org/covid-vaccinations.).

The need to evaluate revaccination schemes rose because of the emergence of viral variants escaping the immune response as consequence of SARS-CoV-2 evolution, which provides adaptation and increased fitness. SARS-CoV-2, alike other RNA coronaviruses, has proofreading activity at its exoribonuclease ExoN (6, 7) leading to mutation rates estimated in around 10^-6^ and 10^-7^ per site per replication cycle (8), considerably lower than in other RNA viruses. Despite this low mutation rate, advantageous mutations with respect to viral replication, transmission, and immune evasion are selected in natural populations (9). Accumulation of such mutations in certain genotypes leads to the rise of the so-called variants of concern (VOC). Many of these mutations are located in the S protein, which contains two domains, namely S1 and S2. The N-terminal region of the S1 domain contains the species-specific receptor-binding domain (RBD). Mutations in this domain have been detected in circulating variants (10). In addition to the increase in contagiousness, an increasing immune evasion with Beta (B.1.351), Delta (B.1.617.2), and now Omicron (B.1.529 and its sub-linages) VOC were detected (11–13). These new properties of SARS-CoV-2 have contributed to a decreased in vaccine effectiveness and in the duration of the conferred protection (14, 15).

To accelerate the path toward modified vaccines or new vaccines, the knowledge on the correlate of protection for COVID-19 and surrogate immunological markers are necessary. Although no consensus has been reached, analysis of SARS-CoV-2 antibodies from participants in COVID-19 vaccine efficacy trials has brought scientists closer to identifying a correlate of protection against COVID-19. In this sense recently in a coronavirus efficacy phase 3 trials designated COVE trial was found that several neutralizing and binding antibody markers, including the 50% inhibitory dilution (ID50) neutralizing antibody titers, were correlated with the efficacy of the vaccine for 4 months (16). Participants with ID50 neutralization titers of 10, 100, and 1,000 had an estimated vaccine efficacy of 78%, 91%, and 96%, respectively, against laboratory-confirmed, symptomatic COVID-19 (16). Though replication in other phase 3 trials is needed to further strengthen the evidence, the results are robust as this was a randomized, double-blind, placebo-controlled trial.

In this work, we present the preclinical results on the immunogenicity of vaccine candidates employing protein-based vaccine platform containing the trimeric and glycosylated S of different SARS-CoV-2 VOC adjuvanted with alhydroxyaluminum.

## Materials and methods

### Recombinant S protein from different SARS-CoV-2 variants

Recombinant Spike trimer constructs are based on “tagless” versions of the SARS-CoV-2 Spike trimers described previously (17, 18). Briefy, SARS-CoV-2 sequences (Genbank accession number MN908947) were codon-optimized for Chinese Hamster Ovary (CHO) cells and synthesized by GenScript. Within the construct, the spike glycoprotein was preceded by its natural N-terminal signal peptide and fused at the C-terminus to the foldon domain of the T4 phagehead fibritin (19). Mutations were added to stabilize the generated spike protein in the pre-fusion conformation (K986P–V987P) and the furin site was abolished (RRAR^682–685^ –GGAS) as previously described (20). The D614G mutation was also inserted in the Wuhan ancestral variant. Constructs were then cloned into pTT241 plasmid that did not encode C-terminal FLAG/His affinity tags. Expression constructs for VOC Spike variants were prepared as previously described. Intranasal immunization with a proteosome-adjuvanted SARS-CoV-2 spike protein-based vaccine is immunogenic and efficacious in mice and hamsters (21). Ancestral, B and D spike proteins were purified using a one-step affinity method with NGL COVID-19 Spike Protein Affinity Resin (Repligen, Waltham, MA, USA). Purified proteins analyzed by sodium dodecyl sulphate-polyacrylamide gel electrophoresis (SDS-PAGE) and analytical size-exclusion ultra-high performance liquid chromatography (SEC-UPLC) as previously described (18). The identity and purity of the antigens was also confirmed by mass spectrometry. Absence of endotoxin contamination was verified using Endosafe cartridge-based Limulus amebocyte lysate tests (Charles River Laboratories, Charleston, SC, USA).

### Mice

BALB/c female mice with 5 to 6 weeks of age, (School of Veterinary Sciences, La Plata, Argentina) were randomly divided into immunized and non immunized groups with 6 to 8 mice in each group. All procedures were performed in a BSL-2 facility with approval from Ethical Committee for Animal Experiments of the Faculty of Science at La Plata National University (Argentina, approval number 010-38-21 y 004-40-22).

### Immunization and sample collection

Spike proteins were formulated with alhydrogel 2% (*In vivo*Gen, San Diego, CA, USA). Protein adsorption was analyzed by sodium dodecyl sulphate–polyacrylamide gel electrophoresis, followed by Coomassie staining. Protein concentration was determined by the Bradford method. Vials of Gam-COVID-Vac-rAd26/rAd5, AZD1222/ChAdOx1, BBIBP-CorV were acquired from Ministry of Health of Buenos Aires province. Female BALB/c mice (*n* = 8) were immunized (i.m) with 2 doses or 2-dose primary schedule plus 1 dose of booster at days 0 and 14 or 0, 14 and 49, respectively. Blood was collected from isoflurane-anesthetized mice, *via* the submandibular vein on day 14-post vaccination dose. All mice were humanely euthanized by cervical dislocation to collect spleens.

### Anti-spike IgG ELISA

Anti-spike total IgG titers and IgG isotypes in serum were quantified by ELISA. Briefly, 96-well high-binding ELISA plates (Nunc A/S, Roskilde, Denmark) were coated overnight at 4°C with 100 µL of 0.45 µg/mL Spike protein (same as used for immunization). Plates were washed five times with PBS/0.05% Tween20 (PBS-T), and then blocked for 1 h at 37 °C with 200 µL 3% milk in PBS before incubation with serially diluted samples of mouse serum (1 h, 37°C). After five washes with PBS-T (Sigma-Aldrich), 100 µL of horseradish-peroxidase–labeled goat anti-mouse IgG (Invitrogen, United States) at 1:8,000. For measuring the IgG isotypes, the bound antibody was incubated with horseradish-peroxidase labeled subclass-specific anti-mouse IgG1 at 1:8,000 or anti-mouse IgG2a at 1:1,000 (Sigma, Aldrich). After five washes with PBS-T, 100 µL/well of the substrate o-phenylenediamine dihydrochloride (OPD, Sigma-Aldrich) diluted in 0.05 M citrate buffer (pH 5.0) was added. Plates were developed for 15 min at RT in the dark. The reaction was stopped with 50 µL/well of 4 N H2SO4. Bound IgG Abs were detected spectrophotometrically at 492 nm and then plotted as a function of the log of the (serum dilution)^–1^. A successful assay for each antibody sample produced a sigmoidal curve in this type of plot. The titer of each antibody sample was determined from this curve by identifying by GraphPad Prism^®^ software the concentration (expressed as inverse of the dilution of the antibody) that provokes a half way signal between the basal response and the maximal response. The cut-off levels determined for IgG, IgG1, and IgG2a assays were 12.5 ± 3.6, 1.9 ± 0.9, and 5.8 ± 2.7, respectively.

### Pseudovirus neutralization assay

Neutralization assays were carried out with SARS-CoV-2 pseudotyped particles (CoV2pp-GFP from Sean Whelan laboratory) (22) that carries vesicular stomatitis virus as viral backbone and expresses full length wild-type spike from ancestral or B, D or Omicron variant on its envelope.

Viral stocks (VSV-eGFP-SARS-CoV-2) were amplified using 293T ACE2/TMPRSS2 cells at an MOI of 0.01 in Dulbecco’s Modified Eagle’s medium containing 2% FBS at 37°C. Viral supernatants were harvested upon extensive cytopathic effect and GFP positive cells. The media was clarified by centrifugation at 1,000 x g for 5 min. Viral stocks were titrated by fluorescence forming units per milliliter (UFF/ml) in Vero cell line. Aliquots were maintained at -80°C.

For neutralization assays, Vero cells maintained with DMEM high glucose with 10% FBS were seeded in a 96-well plate the day before infection. Mice sera were heat inactivated at 56 °C for 30 minutes and serially diluted in DMEM high glucose medium. Serum neutralizations were performed by first diluting the inactivated sample 2-folds and continuing with a 2-fold serial dilution. A pre-titrated amount of pseudotyped particles was incubated with a 2-fold serial dilution of patient sera for 1 h at 37°C prior to infection. Subsequently, cells were fixed in 4% formaldehyde containing 2 mg/mL DAPI nuclear stain (Invitrogen) for 1 hour at room temperature, and fixative was replaced with PBS. Images were acquired with the InCell 2000 Analyzer (GE Healthcare) automated microscope in both the DAPI and FITC channels to visualize nuclei and infected cells (i.e., eGFP-positive cells), respectively (4X objective, 4 fields per well, covering the entire well). Images were analyzed using the Multi Target Analysis Module of the InCell Analyzer 2000 Workstation Soft-ware (GE Healthcare). GFP-positive cells were identified in the FITC channel using the top-hat segmentation method and subsequently counted within the InCell Workstation software. Absolute inhibitory concentrations (absIC) values were calculated for all animals sera samples by modeling a 4-parameter logistic (4PL) regression with GraphPad Prism 9.00. Absolute inhibitory concentration was calculated as the corresponding point between the 0% and 100% assay controls. Fifty % inhibition was defined by the controls for all the samples on the same plate.

### Ag-specific IL-17 and IFN-γ production by spleen cells and FACS analysis

Spleens from untreated and immunized mice were passed through a 40-mm cell strainer to obtain a single-cell suspension. Spleen cells were cultured with spike protein (S: 1µg/ml), or medium only. After 72 h of incubation, IFN-γ and IL-17A concentrations were quantified in supernatants by ELISA.

For FACS analysis, spleen cells that were not re-stimulated with Spike protein were incubated with CD16/CD32 FcgRIII (1:100) to block IgG Fc receptors. Cells were treated with LIVE/DEAD Violet (Invitrogen), followed by surface staining with fluorochrome-conjugated anti-mouse Abs for various markers: CD3-Pe-Cy7 (BD), CD8-PE (BD), CD4-FITC (BD), CD44-PE (BD), CXCR5-PerCP-Cy5.5 (BD), CD127-APC (BD). Flow cytometry analysis was performed on an BD FACSAria Fusion. The results were analyzed using FlowJo software (TreeStar).

### Statistical analysis

The data were evaluated statistically by a one-way analysis of variance (ANOVA) followed by the Tukey test *post hoc* or Bonferroni´s multiple comparison test as appropriate. For fluorescence values analysis, we used Mann-Whitney statistical analysis. Differences were considered to be significant when p< 0.05. All statistical analysis of data was performed using GraphPadPrism^®^ version 9.00 for Windows, GraphPad^®^ Software.

## Results

### Spike protein expression and purification

**Figure 1:**
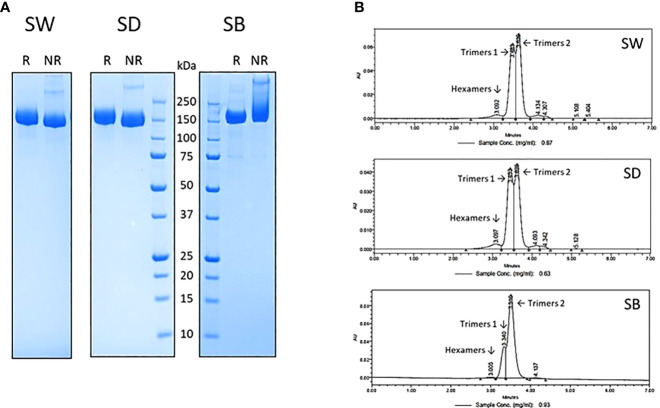
SARS-CoV-2 trimeric spike protein variants were produced in stable CHO pool and purified using a proprietary 3-step chromatographic process and analyzed by reducing (R) and non-reducing (NR) SDS-PAGE **(A)** and analytical size exclusion chromatography **(B)** as described in materials and methods. All spikes elute from the SEC-UPLC column as a mixture containing various amounts of Hexamers, Trimers 1 and Trimers 2 species. SW: ancestral Wuhan spike; SD: Delta spike variant (B.1.617.2); SB: Beta spike variant (B.1.351).

The tag-less ancestral Wuhan-D614G, B.1.617.2 and B.1.351 (SW, SD and SB) trimeric spike proteins were expressed from stable CHO pools as described previously (23). Their purification to near homogeneity was achieved using a proprietary in-house 3-step chromatographic process, including a low pH-hold viral inactivation step. Their purity, as assessed by reducing and non-reducing SDS-PAGE (**Figure 1A**) and analytical size-exclusion chromatography (SEC-UPLC; **Figure 1B**), was estimated at >95%. The SEC-UPLC was linked to a MALS detector and the apparent mass of the 3 spike proteins was of ~520-650 kDa for both “trimers 1” and “trimers 2” (data not shown). The spikes were all eluting as a mixture of “trimers 1” and “trimers 2” plus a low amount of hexamers (trimeric and hexameric species confirmed by MALS analyses). We consistently observed the presence of trimers 1 and trimers 2 in various spike preparations and their resolution by SEC-UPLC is suggestive of substantial conformational differences between the two forms. The reason(s) behind those structural differences however remain currently unknown.

### Glycosylated trimeric protein S formulations dose-response assays

**Figure 2:**
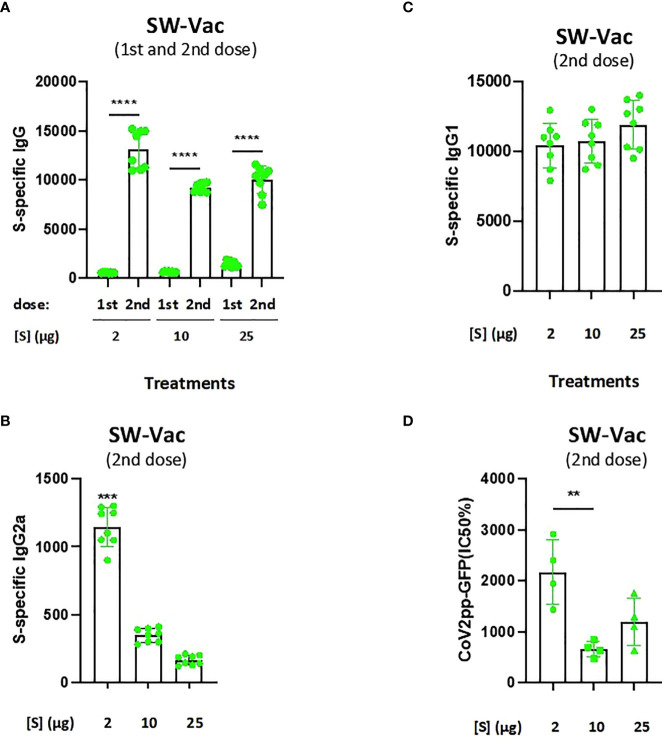
Anti-spike IgG titers and neutralization titers induced by SW-Vac formulations in mice. BALB/c mice (n = 8/group) were immunized twice on Days 0 and 14 with SW-Vac formulations containing 2, 10 o 25µg of purified SW protein delivered intramuscularly. Serum collected on Day 14 was analyzed by ELISA to determine the levels of S-specific IgG **(A)** and IgG isotypes (**B** and **C** titers). For statistical analysis, antibody titers were log-transformed and then analyzed by a one-way ANOVA with Bonferroni’s multiple comparisons test. ***p < 0.001, ****p < 0.0001. Neutralizing titers were measured by 50% inhibition for the pseudotyped virus (CoV2pp-GFP) in immunized and non-immunized mice **(D)**. Absolute inhibitory concentration was calculated as the corresponding point between the 0% and 100% assay controls. Fifty % inhibition was defined by the controls for all the samples on the same plate. For statistical analysis, values were analyzed by a one-way ANOVA with Bonferroni`s multiple comparisons test. **p < 0.01, n.s. no significant difference.

The purified ancestral Wuhan spike protein with the D614G mutation (SW) was formulated with alhydrogel adjuvant. Groups of BALB/c mice (n=8) were vaccinated two times with doses from 2, 10 and 25µg of SW-Vac. IgG, IgG1 and IgG2a titers against SW protein (a-SW) were measured after vaccination. In all cases, immunoglobulin titers of the immune sera were obtained by plotting the absorbances at 492 nm vs the log of the reciprocal of the dilution in the GraphPad Prism program. The titers are expressed as the inverse of the dilution of the inflection point of the curve. **Figure 2A** shows that all immunized mice induced a SW-specific IgG response (IgG a-SW) after both the first and second doses, with titers being higher after the second dose, regardless of the quantity of the protein S used in the formulation. After the second dose, no significant differences were detected among the IgG a-SW titers obtained for each of the 3 amounts of S tested. It is important to highlight that the schedule containing the S protein at 2µg (SW-Vac 2µg) triggered murine antibody responses with the highest IgG2a/IgG1 (**Figures 2B, C**). The IgG2a/IgG1 ratio detected for the SW-Vac 2µg treatment suggests that this dose skewed the immune response to a Th1/Th2 mixed profile. Neutralizing capacity was also evaluated by measuring antibody-neutralizing titers against W ancestral variant for immunized and non-immunized animals, using the CoV2pp-GFP pseudovirus infection (**Figure 2D**). At 14 days after administration of the last dose, neutralizing antibodies were detected in immunized mice at any of the S-protein amount tested, being the titers detected for SW-Vac 2µg significantly higher than those induced by the formulation SW-Vac 10µg (p<0.01). In contrast, in non-immunized animals, the levels of neutralizing antibodies were undetectable (not shown).

**Figure 3:**
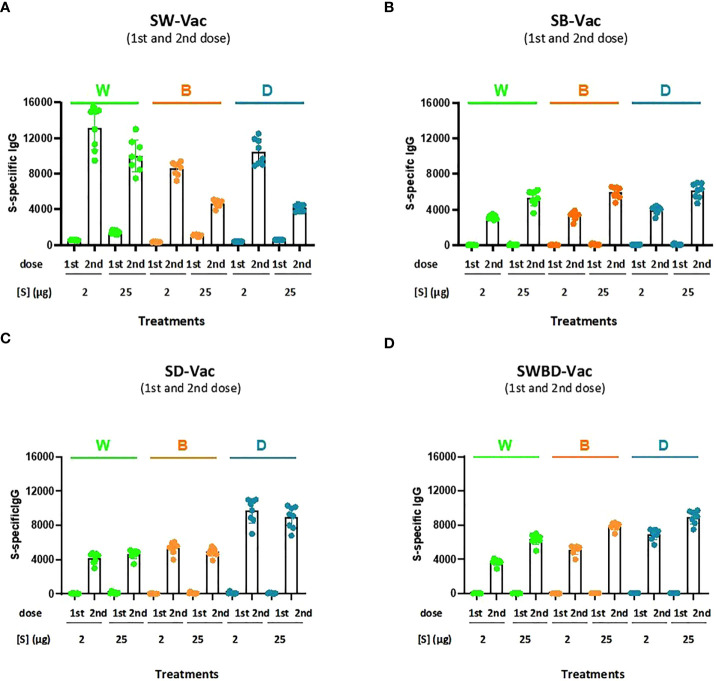
Anti-spike IgG titers induced by S-Vac formulations in mice. BALB/c mice (n = 8/group) were immunized on Days 0 and 14 with formulations containing spike purified protein from ancestral Wuhan **(A)**, Beta (SB, **B**), Delta (SD, **C**) or a combination of spike protein from W, B and D SARS-CoV-2 variants (SWBD, **D**) in 2 different concentrations- 2 and 25 µg delivered intramuscularly. Serum collected on Day 14 was analyzed by ELISA to determine the levels of S-specific IgG. The IgG specificity of the sera was indicated in the top of the figures. Sera titers were obtained by plotting the OD 492 nm vs. log curve of the reciprocal of the dilution in the GraphPad Prism program. Titers are expressed as the inverse of the inflection point dilution of the curve. For statistical analysis, antibody titers were log-transformed and then analyzed by a one-way ANOVA with Bonferroni’s multiple comparisons test. For all treatments, the titers detected after the second dose were significantly different from those found after the first dose (p<0.0001).

Formulations containing spike purified protein from Beta (SB), Delta (SD) or a combination of spike protein from W, B and D SARS-CoV-2 variants (SWBD, with the same proportion of each VOC until reaching the final protein amount to be used, which is 2µg and 25µg) in 2 different final protein amount-2 and 25 µg- were also tested and compared with SW-Vac 2µg or SW-Vac 25µg in the murine model. IgG titers, using ELISA assays to detect antibodies against each of the recombinant S proteins SW, SB (a-SB) or SD (a-SD), were measured after all vaccination schedule tested (**Figure 3**). The **Figure 3** shows that there is cross-reaction among the different tested SARS-CoV-2 variants, since the sera from the groups of animals immunized with each of the variants were able to recognize all three SARS-CoV-2 proteins tested (**Figures 3A–C**). An interesting observation was the detection of higher a-SW, a-SB, a-SD-IgG titers in the sera of mice immunized with SW-Vac 2µg in comparison with those induced in mice immunized with any of the other formulations, even those containing the highest amount of protein tested (**Figure 3**). The highest IgG titers against W, B, and D SARS-CoV-2 S protein variants were detected in mice immunized with SW-Vac 2µg compared to the other treatments (p<0.01). Regarding the SWBD formulation, the IgG titers against the S protein of the 3 SARS-CoV-2 variants were also lower than those obtained with the SW-Vac 2µg formulation (**Figure 3D**). The results obtained with SW-Vac 2µg were in agreement with previous reports on dose ranging data from small animal studies suggesting that immunogenicity of vaccines were the highest at middle doses, decreasing with higher doses (24–26).

**Figure 4:**
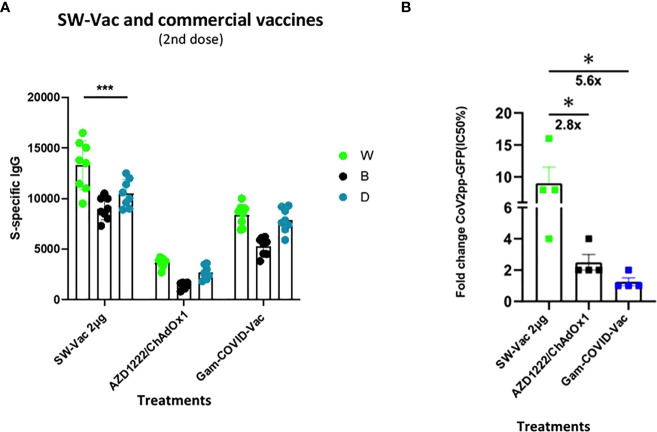
Anti-spike IgG titers and neutralization titers induced by S-Vac formulation and commercial vaccine in mice. BALB/c mice (n = 8/group) were immunized on Days 0 and 14 with formulations containing spike purified protein from ancestral Wuhan (2µg) or commercial Gam-COVID-Vac-rAd26/rAd5 or AZD1222/ChAdOx1 vaccines delivered intramuscularly. Serum collected on Day 14 was analyzed by ELISA to determine the levels of S-specific IgG. Sera titers were obtained by plotting the OD 492 nm vs. log curve of the reciprocal of the dilution in the GraphPad Prism program. Titers of Panel **(A)** are expressed as the inverse of the inflection point dilution of the curve. For statistical analysis, antibody titers were log-transformed and then analyzed by a one-way ANOVA with Bonferroni’s multiple comparisons test. For all treatments, the titers detected after the second dose were significantly different from those found after the first dose (p<0.0001). Neutralizing titers were measured by 50% inhibition for the pseudotyped virus (CoV2pp-GFP) in immunized and non-immunized mice **(B)**. Absolute inhibitory concentration was calculated as the corresponding point between the 0% and 100% assay controls. Fifty % inhibition was defined by the controls for all the samples on the same plate. Data in panel **(B)** is presented as the fold change of CoV2pp-GFP related to the minimum value detected in immunized animals. For statistical analysis, one-way ANOVA with Bonferroni`s multiple comparisons test was used. *p < 0.05; ***p<0.001.

In order to compare the immunogenicity of SW with approved vaccines, the total IgG levels against SW, SB and SD induced by the SW-Vac 2µg formulation were compared to those achieved with commercial Gam-COVID-Vac-rAd26/rAd5 (Gam-COVID-Vac x 2 doses) or AZD1222/ChAdOx1 (AZD1222/ChAdOx1 x 2 doses) vaccines. Application of two doses of SW-Vac 2µg showed significantly higher levels of S-specific IgG than those elicited by 2 doses of Gam-COVID-Vac or AZD1222/ChAdOx1 (**Figure 4A**). In accordance with these results, the neutralizing antibodies against SW ancestral pseudovirus induced by SW-Vac 2µg was 2.8 and 5.6 times higher than those induced by 2 doses of Gam-COVID-Vac or AZD1222/ChAdOx1, respectively (**Figure 4B** p<0.05).

### Cellular immune response induces by SW-Vac 2µg formulation

**Figure 5:**
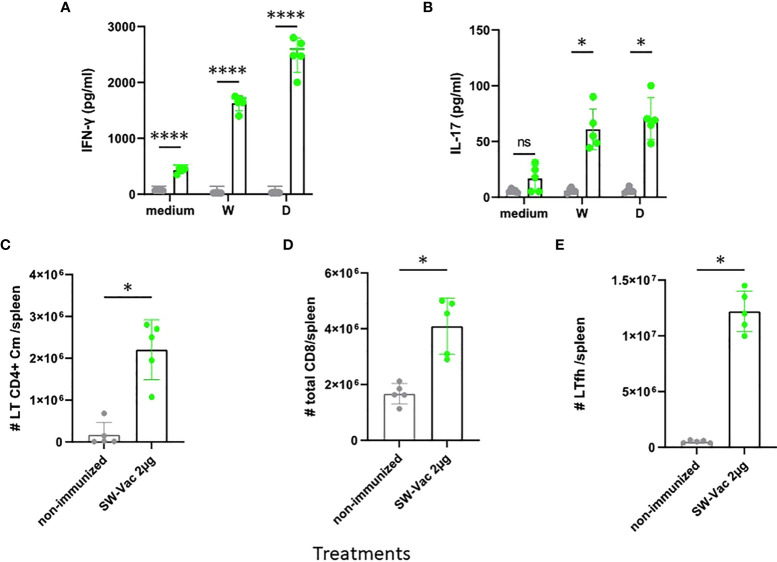
SW-Vac 2µg formulation induces Ag-specific Th1(IFN-γ)/Th17 (IL-17), Tfh and CD8+ T cells in the spleen. BALB/c mice were vaccinated as described in previous figures. Mice were sacrificed 44 days after the second immunization to obtain spleens, and T-cell response was evaluated. Levels of secreted IFN-γ **(A)** and IL-17 **(B)** following splenocytes stimulation with medium or recombinant spike protein from W o D variants were determined by ELISA. Bars are means ± SEM of pg/ml of IFN-γ and IL-17. *p<0.05, ****p<0.0001 t-test. For FACS analysis the spleen cells from immunized mice (green circles) and non-immunized (grey circles) were incubated with CD16/CD32 FcγRII (1:100) to block IgG Fc receptors. Cells were incubated with LIVE/DEAD Violet (Invitrogen), followed by surface staining with fluorochrome-conjugated anti-mouse Abs for various markers: CD3-Pe-Cy7 (BD), CD8-PE (BD), CD4-FITC (BD), CD44-PE (BD), CXCR5-PerCP-Cy5.5 (BD), CD127-APC (BD); CD4^+^Cm: CD3^+^CD4^+^CD62L^+^CD44^+^ **(C)**; CD8^+^: CD3^+^CD8^+^ **(D)**; Tfh: CD3^+^CD4^+^CXCR5^+^ **(E)**. Flow cytometry analysis was performed on an BD FACSAria Fusion. The results were analyzed using FlowJo software (TreeStar).*p<0.05 t-test. Results are representative of two independent experiments.

We then evaluated T-cell-mediated immune responses. To this aim, splenocytes cells from SW-Vac 2µg immunized and non-immunized mice were stimulated with SW, SD or medium alone and then, cytokines levels in the supernatants were measured. Immunization with SW-Vac 2µg induced IFN-γ and IL-17 levels significantly higher (p<0.0001 and p<0.01 respectively) than that detected in supernatants of splenocytes from non-immunized animals (**Figures 5A, B**). Furthermore, IFN-γ and IL-17 levels were detected in the immunized animals when the stimulations were performed with the SARS-CoV-2 variants not included in the formulation. Importantly, the levels of IFN-γ were higher than that of IL-17 at the spleens of immunized mice. In the spleens, immunization with SW2 also promoted a significant increase of central memory T CD4, CD8 and Tfh cells population (**Figure 5**, p<0.05 vs non-immunized mice).

### Use of SW-Vac 2µg formulation as a booster vaccine (third dose)

**Figure 6:**
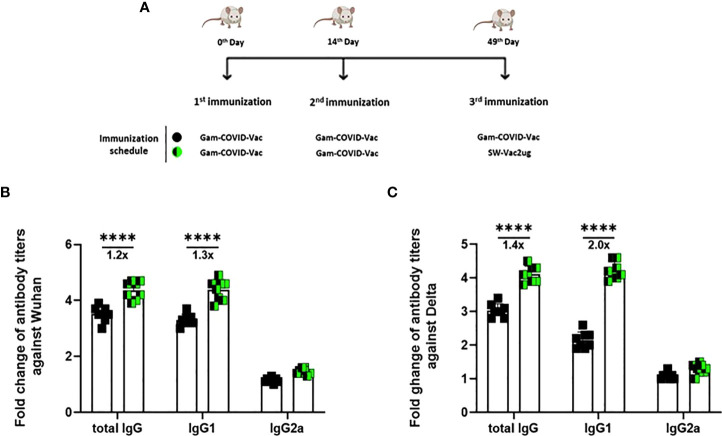
Anti-spike IgG titers induced by a booster with SW-Vac 2µg formulation or commercial vaccine in mice. BALB/c mice (n = 8/group) were immunized on Days 0 and 14 with commercial Gam-COVID-Vac-rAd26/rAd5 vaccine plus a booster with SW-Vac formulation or Gam-COVID-Vac-rAd5 on Day 49 delivered intramuscularly **(A)**. Serum collected on Day 14 after the last dose was analyzed by ELISA to determine the levels of W **(B)** or **(C)** S-specific IgG, IgG1 and IgG2a. Sera titers were obtained by plotting the OD 492 nm vs. log curve of the reciprocal of the dilution in the GraphPad Prism program. Titers are expressed as the inverse of the inflection point dilution of the curve. For statistical analysis, antibody titers were log-transformed and then analyzed by a one-way ANOVA with Bonferroni’s multiple comparisons test. For all treatments, the titers detected after the second dose were significantly different from those found after the first dose (p<0.0001).

**Figure 7:**
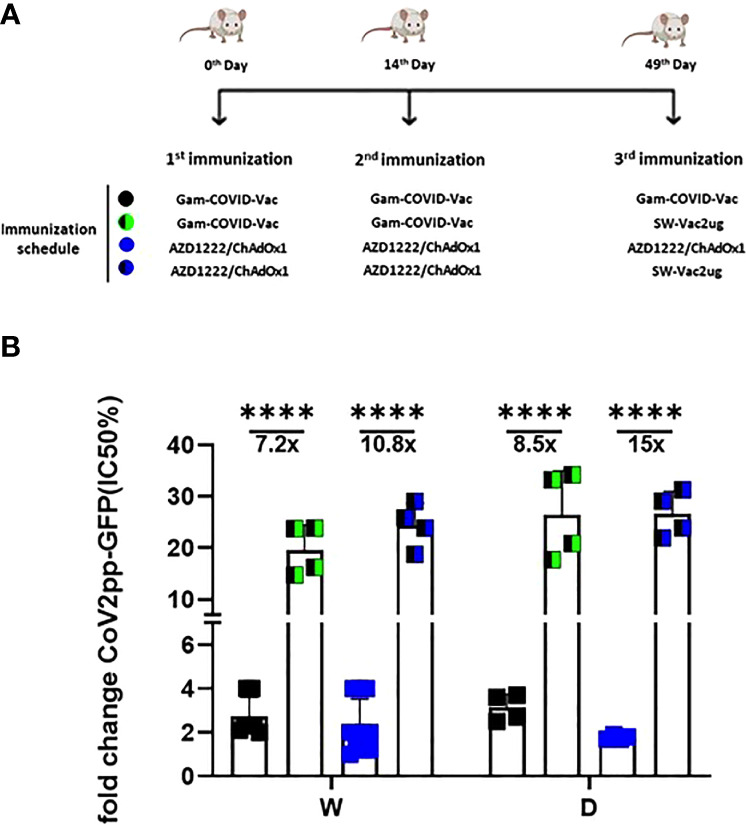
Neutralizing capacity against W or D pseudovirus induced by a booster with SW-Vac 2µg formulation. BALB/c mice were immunized on Days 0 and 14 with commercial Gam-COVID-Vac-rAd26/rAd5 or AZD1222/ChAdOx1 vaccine plus a booster with SW-Vac formulation, Gam-COVID-Vac-rAd5 or AZD1222/ChAdOx1 on Day 49 delivered intramuscularly **(A)**. Serum was collected on Day 14 after the last dose. Neutralizing titers were measured by 50% inhibition for the pseudotyped virus (CoV2pp-GFP) in immunized and non-immunized mice **(B)**. Absolute inhibitory concentration was calculated as the corresponding point between the 0% and 100% assay controls. Fifty % inhibition was defined by the controls for all the samples on the same plate. Data is presented as the fold change of CoV2pp-GFP related to the minimum value detected in immunized animals. For statistical analysis, one-way ANOVA with Bonferroni`s multiple comparisons test was used. ****p < 0.0001.

**Figure 8:**
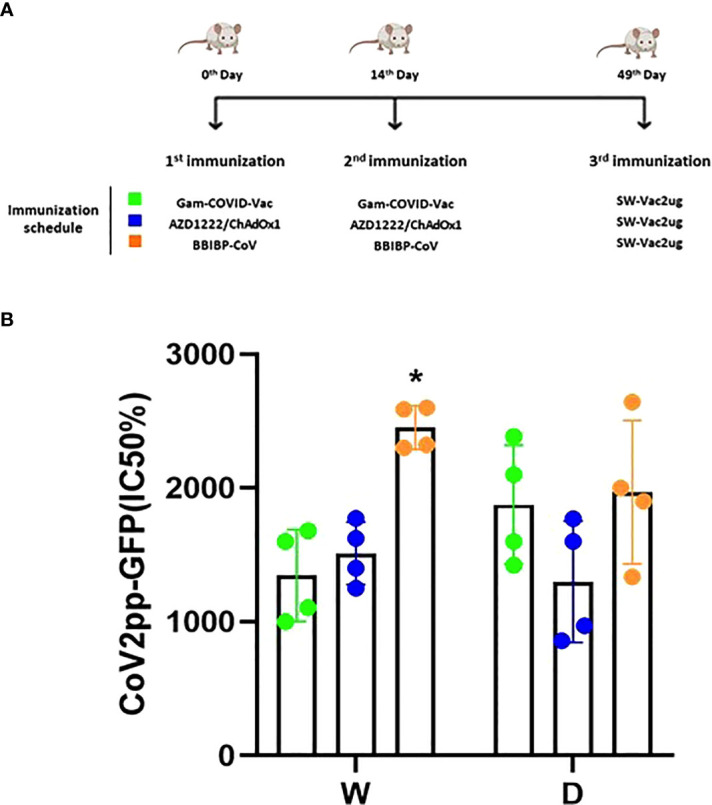
Neutralizing capacity induced by a booster with SW-Vac 2µg formulation. BALB/c mice (n = 4/group) were immunized on Days 0 and 14 with commercial Gam-COVID-Vac-rAd26/rAd5, AZD1222/ChAdOx1 or BBIBPCorV vaccines plus a booster with SW-Vac formulation delivered intramuscularly **(A)**. Serum was collected on Day 14. Neutralizing titers were measured by 50% inhibition for the pseudotyped virus (CoV2pp-GFP) in immunized **(B)**. Absolute inhibitory concentration was calculated as the corresponding point between the 0% and 100% assay controls. Fifty % inhibition was defined by the controls for all the samples on the same plate. For statistical analysis, values were analyzed by a one-way ANOVA with Bonferroni`s multiple comparisons test. *p < 0.05.

Taking into account the need of booster doses to sustain over the time the immunity that could be effective against the different SARS-CoV-2 VOC, we evaluated the ability of SW-Vac 2µg to work as a booster dose after primary immunization with approved vaccines. For this, we carried out vaccination schemes that included 2 doses spaced 14 days apart and a third booster dose applied 35 days after the second dose (**Figure 6A**). For the primary series, 2-dose schemes, SW-Vac 2µg and Gam-COVID-Vac were used. Total IgG titers against SW in both booster schedules with SW-Vac 2µg increased more than 4.5 fold respect to the levels observed in the 2 tested primary schemes (p<0.01, not shown). The same analysis was done against spike protein from the Delta variant. After receiving the booster, anti-SD IgG titers increased at least 2-fold compared to those detected for the 2-dose schemes (p<0.01, not shown). Finally when comparisons were performed between SW-Vac 2µg and Gam-COVID-Vac (rAd26) booster (3rd dose) on a primary scheme of 2 doses of Gam-COVID-Vac, total S-specific IgG levels against SW and SD detected were at least 1.2 times higher in the case of the SW-Vac 2µg 3rd dose (**Figure 6B** p<0.0001). The IgG2a levels against SW and SD variant were indistinguishable for the two 3-dose regimens tested, however for the schedule consisting in 2 doses of Gam-COVID-Vac plus 1 dose of SW-Vac 2µg, the IgG1 levels were at least 1.3 **Figure 6B** and 2 times higher against SW and SD, respectively (**Figures 6B, C** p<0.0001) when compared to those detected in the animals treated with 3-dose schedule of Gam-COVID-Vac. Even more interesting, the SW-Vac 2µg booster induced 7.2 and 8.5 times higher neutralizing antibodies levels against SW and SD, respectively (**Figure 7** p<0.0001) when compared to those induced by Gam-COVID-Vac (rAd26) boost. The impact of SW 2µg as a third dose in inducing humoral response was also evaluated in schedules that used two doses of AZD1222/ChAdOx1 as a primary series. A group of animals that received AZD1222/ChAdOx1 as a as a third dose was also included for comparisons purposes. In this case, the third dose of SW-Vac 2µg showed 10.8 and 15 times higher neutralization antibody titers against SW and SD, respectively, in comparison to those detected with AZD1222/ChAdOx as a third dose (**Figure 7** p<0.0001). Regardless the primary scheme used, Gam-COVID-Vac or AZD1222/ChAdOx1, both non-replicative adenovirus platforms, the neutralizing antibody levels detected after the third dose with SW-Vac 2µg were similar. It is interesting to note that when SW-Vac 2µg was used as third dose after a primary scheme (2 doses) with Sputnik, ChAdOx1 or BBIBP-CorV, the levels of neutralizing antibodies against W were significantly higher with the inactivated virus platform BBIBP-CorV than those detected when the primary scheme was with non-replicative vector platforms (p<0.05, **Figure 8**).

**Figure 9:**
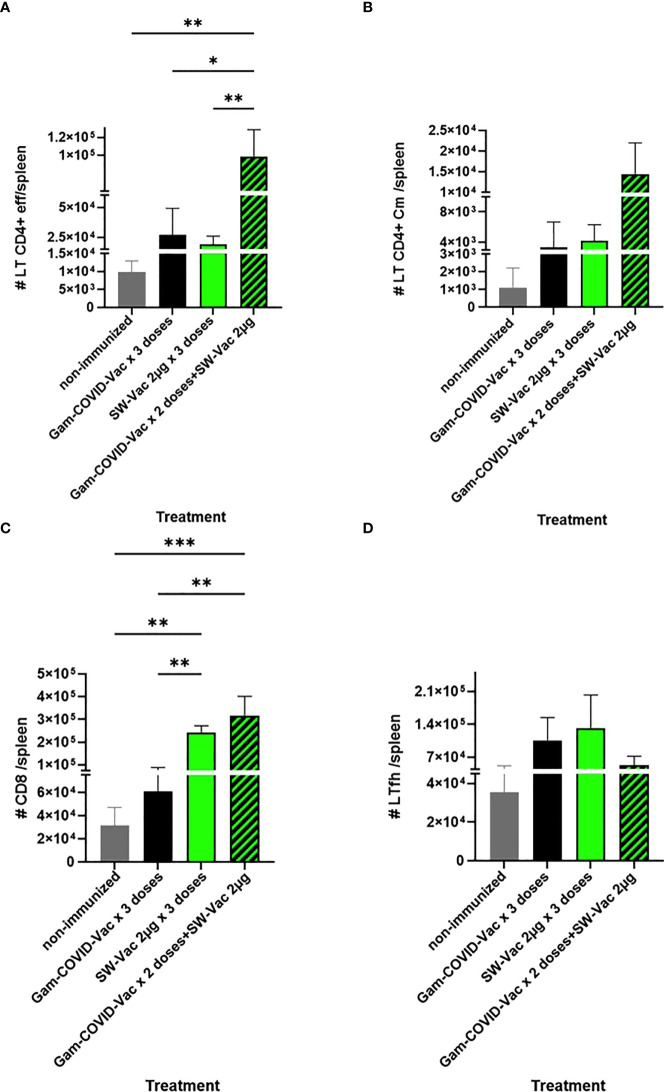
Flow cytometry analysis of spleen cells from immunized (3-doses schedules) or non-immunized mice were incubated with CD16/CD32 FcgRIII (1:100) to block IgG Fc receptors. Cells were incubated with LIVE/DEAD Violet (Invitrogen), followed by surface staining with fluorochrome-conjugated anti-mouse Abs for various markers: CD3-Pe-Cy7 (BD), CD8-PE (BD), CD4-FITC (BD), CD44-PE (BD), CXCR5-PerCP-Cy5.5 (BD), CD127-APC (BD); CD4^+^effector: CD3^+^CD4^+^CD62L^-^CD44^+^ **(A)**; CD4^+^Cm: CD3^+^CD4^+^CD62L^+^CD44^+^ **(B)**; CD8^+^: CD3^+^CD8^+^ **(C)**; Tfh: CD3^+^CD4^+^CXCR5^+^ **(D)** Flow cytometry analysis was performed on an BD FACSAria Fusion. The results were analyzed using FlowJo software (TreeStar).*p<0.05, **p<0.01, ***p<0.001 t-test. Results are representative of two independent experiments.

When SW-Vac 2µg was used as a heterologous booster (third dose) a significant increment of effector memory T CD4 and CD8 cells population in comparison with homologous 3 doses schedules was detected (**Figure 9**). Though an increment in central memory T CD4 and Tfh cells were also detected for the SW-Vac 2µg booster (third dose) in comparison to the homologous 3 dose schedules, this increment was not significant (**Figure 9**).

## Discussion

The purpose of this study was to evaluate the immunogenicity of vaccine candidates based on the recombinant S protein of different SARS-CoV-2 VOC, in particular the ancestral Wuhan, Beta, and Delta variants. Beta and Delta variants share some mutations with other variants including Omicron (27). Beta (B.1.351) was found in South Africa and manifested a rapid regional distribution to an over 80% prevalence (28–30). In May 2021, Delta (B.1.617.2) was first detected in India and rapidly became the dominant variant worldwide by late 2021 in an epidemic that lasted quite long in the world until Omicron (B.1.1.529) was detected in November 2021 (31–33).

One of the most common mutations in the S protein of SARS-CoV-2 virus is the N501Y substitution that, among others, was found in Beta and Omicron (11). This mutation, as well as E484K from Beta or L452R and T478K from Delta were reported to increase the affinity with the ACE2 receptor for viral entry (34–36). These mutations affect the neutralizing capacity of both vaccination-induced antibodies and even recombinant monoclonal antibodies (36–39). Moreover, the detected mutations in SARS-CoV-2 VOC seem to be responsible for the lower effectiveness of the 2-dose schedule compared to that detected against the ancestral virus (40–42). Thus, the introduction of boosters to the primary 2-dose vaccine schedules allowed the recovery of at least part of the vaccine effectiveness in this scenario. Enhanced immunogenicity was particularly detected when heterologous boosters were used (43, 44).

In the medium term, an alternative to improve the effectiveness of current vaccines against the disease caused by circulating VOC is the inclusion of the circulating SARS-CoV-2 variants in the vaccine platforms that are being used (45). In fact, bivalent protein-based or RNA-based vaccines specific to the ancestral strain and a VOC have already been designed, showing balanced neutralizing activities against both strains in the murine model (46, 47). Some of the bivalent vaccine candidates have even entered the clinical stage for further evaluation of efficacy against VOC (NCT04927065, NCT05004181, NCT04889209, NCT05029856, NCT04904549). To face the pandemic caused by VOC, several strategies must undoubtedly be tested, including those that explore the composition not only in terms of immunogenic diversity but also in terms of the quality of the components, their integrity, and molecular structure. In this study, we explored the immunogenicity of vaccine candidates based on the trimeric glycosylated full S protein from different VOC. Our results showed that the SW-Vac containing 2µg of purified S protein from ancestral Wuhan SARS-CoV-2 induced high antigen-binding, and neutralizing antibodies against pseudovirus expressing the S protein from the ancestral Wuhan strain, as well as Th17-, Th1-skewed immune responses (**Figure 5**) and T-CD4+ central memory, CD8 and Tfh cell populations. These detected immune profiles are likely involved in conferring protection (48). SW-Vac 2µg formulation was able even to induce high antigen-binding and neutralizing antibody against Beta and Delta variants (**Figure 3A**). It is interesting to note that the neutralizing capacity against the 3 VOC here tested induced by the 2-dose SW-Vac 2µg scheme was higher to that detected for the commercial Gam-Vac vaccine or AZD1222/ChAdOx 1 (**Figure 4B**). All these results, obtained with the lowest dose assayed (i.e. SW 2µg) were in agreement with those reported on dose-ranging data from small animal studies suggesting that the immunogenicity of the vaccine was highest at middle doses and then decreased with the higher doses (24–26). A suggested explanation for this peaked or n-shaped dose-response curve in the context of TB immunogenicity (49), is that after a higher vaccine dose, T cells tend more toward an exhaustive state, i.e. increased differentiation into a terminal state (50). Similar peaked dose-response curves in which smaller doses were more immunogenic than higher doses were also observed in clinical trials (51, 52).

Recombinant S protein or its parts (including RBD, S1, S2, trimerized RBD, full length spike ectodomain, etc.) have been evaluated as candidate vaccines in different animal models, at different concentrations, different schedules and formulated with different adjuvants (53–60). Beyond experimental differences, in all cases the results obtained show that the different versions of S protein have immunostimulatory capacity to some degree. Though comparisons are difficult to establish due to the diversity of vaccine formulations assayed, it is noteworthy that a dose as low as 1µg of the full length spike ectodomain formulated with alhydrogel was immunogenic in the murine model not only in terms of protein S-specific antibody levels but also in neutralizing antibody titers (53).

Cross-strain immunity against Wuhan and Beta was also observed for the formulation containing the S protein from Delta variant at 2µg. This result is in agreement with a recent report showing that Delta infection induced a cross-variant neutralization of different SARS-CoV-2 variants (61).

Because at least half of the world population have received 2 doses of a COVID-19 vaccine, we assessed the use of the SW-Vac 2µg formulation as a booster (third dose). Immunogenicity of mice receiving 2 doses of commercial vaccine Gam-COVID-Vac and boosted with SW-Vac 2µg formulation was evaluated in comparison with schedules consisting in 3 doses of either Gam-COVID-Vac (Gam-COVID-Vac with rAd26 used as a booster) or SW-Vac 2µg. Our results showed that all animals receiving a third dose enhanced the IgG and IgG1 levels against Wuhan and Delta, in comparison with those receiving the 2-dose schedules (p<0.05). Higher increases in IgG levels and titers of neutralizing antibodies against SARS-CoV-2 pseudotyped particles expressing S from ancestral Wuhan or Delta variants were also detected for the SW-Vac 2µg booster compared to the Gam-COVID-Vac (rAd26) boost (p<0.0001 **Figure 7**). Similar results were observed when comparisons were performed between SW-Vac 2µg booster vs AZD1222/ChAdOx1 booster on 2-dose AZD1222/ChAdOx1 series (p<0.00001, **Figure 7**) or with the inactivated virus platform BBIBP-CorV (p<0.05, **Figure 8**). The cross-reactive immunity mediated by heterologous boosting as here performed was also detected in recent studies using heterologous Omicron vaccine-boosted mouse or non-human primate models (62–64).

Taken together, the results obtained here show that the vaccine platforms containing the glycosylated trimeric spike protein could be considered as candidates with great potential for use in primary schemes as well as in heterologous boosters. In fact, the use of formulations containing the S protein from the ancestral virus as a booster against Omicron and its sub-lineages was supported by previous studies (65). Though Omicron sub-lineages are able to evade polyclonal neutralizing antibody responses elicited by the primary vaccine series (66, 67), boosters have been shown to be effective against Omicron-induced severe disease. Particularly, it was recently shown that the Wuhan-Hu-1 spike sequence markedly increased neutralizing antibody titers against the BA.1, BA.2, BA.2.12.1 and BA.4/5 Omicron sub-lineages (65).

## Data availability statement

The original contributions presented in the study are included in the article/supplementary material. Further inquiries can be directed to the corresponding author.

## Ethics statement

The animal study was reviewed and approved by Ethical Committee for Animal Experiments of the Faculty of Science at La Plata National University (Argentina, approval number 010-38-21 y 004-40-22).

## Author contributions

DH planned the study, made the laboratory analysis, interpreted data, perform some experiments and drafted manuscript. AW and AG planned the study, interpreted data, and revised figures and the manuscript. MS purified the S proteins. YD expressed and purified the S proteins, revised figures and the manuscript. DB, MGLL and EZ performed certain experiments, interpreted data, and revised figures and the manuscript. PMA and ER performed experiments and laboratory analyses. MGLL performed pseudovirus neutralization assays. All authors approved the final manuscript.

## Funding

This work was supported by ANCPyT (FORNASEC 05/2021), and UNLP (Argentina) grants to AW, AG, and DH, and DH respectively. AW, AG, DB, DH, EZ and EG, are members of the Scientific Career of CONICET. PMA, ER re CONICET fellows.

## Acknowledgments

We would like to acknowledge members of the Mammalian Cell Expression Section of the NRC-HHT for their contribution at producing, purifying and analyzing the spike proteins used in this study. Luciana Cayuela provided excellent technical assistance.

## Conflict of interest

The authors declare that the research was conducted in the absence of any commercial or financial relationships that could be construed as a potential conflict of interest.

## Publisher’s note

All claims expressed in this article are solely those of the authors and do not necessarily represent those of their affiliated organizations, or those of the publisher, the editors and the reviewers. Any product that may be evaluated in this article, or claim that may be made by its manufacturer, is not guaranteed or endorsed by the publisher.
